# Impact of Abnormal Phosphorylation on the Oligomerization of Tau Repeat R2

**DOI:** 10.1002/alz.084379

**Published:** 2025-01-03

**Authors:** Viet H Man

**Affiliations:** ^1^ University of Pittsburgh School of Pharmacy, Pittsburgh, PA USA

## Abstract

**Background:**

Reversible post‐translational modifications, phosphorylation and dephosphorylation, on tau protein play a critical role in the microtubule (MT) modulation. However, abnormal tau phosphorylation, which occurs in tauopathies such as Alzheimer’s disease (AD), causes the dissociation of tau from MTs. The dissociated tau then aggregates into sequent forms from soluble oligomers to paired helical filaments (PHF), and insoluble neurofibrillary tangles (NFTs), a hallmark of AD. In this study, we examine the impact of abnormal phosphorylation on monomeric and dimeric structures of tau repeat R2 peptides, which is the essential segment for tau fibrillization and somehow can represent the full‐length tau.

**Methods:**

Wild‐type tau repeat R2 (wtR2) was taken from an experimental structure of R2‐microtubule complex (PDB code 6CVN). The phosphorylated Ser289 R2 peptide (pS289R2), phosphorylated Ser293 R2 peptide (pS293R2), and phosphorylated Ser289 and Ser293 R2 peptide (pS289+pS293R2) were obtained by phosphorylating the wtR2 at Ser289 and/or Ser293 residues. Extensive replica exchange molecular dynamics simulations using all‐atom force field and explicit solvent were carried out. The structures of R2 monomers and dimers sampled by the simulations were characterized and compared.

**Results:**

For monomeric conformations, the phosphorylation enhances intramolecular interaction, compactness and β content. For dimeric cases, the phosphorylation increases intermolecular interaction, β‐sheet formation and turn‐coil structural transition. Interestingly, the phosphorylated residue strongly interacts with cations, resulting in a pSer‐cation‐pSer bridge.

**Conclusion:**

Overall, our results suggest that abnormal phosphorylation at Ser289 and/or Ser293 accelerates the oligomerization of R2 peptides, and consequently it may promote tau aggregation. Furthermore, we also found a pSer‐cation‐pSer bridge formed, thus uncovering the essential role of cation ions in the oligomerization of the phosphorylated R2 repeat. Our findings suggest that phosphorylation at Ser289 and/or Ser293 should be taken into consideration when screening the inhibitors of tau oligomerization.

